# An Accurate Linear Method for 3D Line Reconstruction for Binocular or Multiple View Stereo Vision

**DOI:** 10.3390/s21020658

**Published:** 2021-01-19

**Authors:** Lijun Zhong, Junyou Qin, Xia Yang, Xiaohu Zhang, Yang Shang, Hongliang Zhang, Qifeng Yu

**Affiliations:** 1 School of Aeronautics and Astronautics, Sun Yat-Sen University, Guangzhou 510275, China; zhonglj9@mail.sysu.edu.cn (L.Z.); qinjy6@mail2.sysu.edu.cn (J.Q.); yangxia7@mail.sysu.edu.cn (X.Y.); 2 College of Aerospace Science and Engineering, National University of Defense Technology, Changsha 410003, China; shangyang1977@nudt.edu.cn (Y.S.); hl_zhang_2000@hotmail.com (H.Z.); yuqifeng@vip.sina.com.cn (Q.Y.)

**Keywords:** plane intersection, 3D line reconstruction, Point-then-Direction, corresponding image points intersection, linear solution

## Abstract

For the problem of 3D line reconstruction in binocular or multiple view stereo vision, when there are no corresponding points on the line, the method called Direction-then-Point (DtP) can be used, and if there are two pairs of corresponding points on the line, the method called Two Points 3D coordinates (TPS) can be used. However, when there is only one pair of corresponding points on the line, can we get the better accuracy than DtP for 3D line reconstruction? In this paper, a linear and more accurate method called Point-then-Direction (PtD) is proposed. First, we used the intersection method to obtain the 3D point’s coordinate from its corresponding image points. Then, we used this point as a position on the line to calculate the direction of the line by minimizing the image angle residual. PtD is also suitable for multiple camera systems. The simulation results demonstrate that PtD increases the accuracy of both the direction and the position of the 3D line compared to DtP. At the same time, PtD achieves a better result in direction of the 3D line than TPS, but has a lower accuracy in the position of 3D lines than TPS.

## 1. Introduction

The 3D line reconstruction is a basic problem in computer vision and optical measurements. It is widely used in computer vision problem, such as 3D scene reconstruction, non-cooperative target reconstruction and pose estimation for symmetric targets. Line features in the image are robust, stable, and easy to extract; the corresponding lines and points are often used in 3D reconstruction. In the position and attitude estimation for the non-cooperative target, the non-cooperative target is often reconstructed by points, lines, rectangles, or other shapes composed of lines, then the reconstructed model is used for tracking and relative pose estimation in subsequent frames. In pose estimation for the long symmetrical target, such as rockets and missiles, the direction of the target’s central axis is often used to represent the target’s attitude, which is often reconstructed by the Direction-then-Point (DtP) method. From the above analysis, we can determine that the accuracy of the 3D line is of significance for computer vision.

There are many studies on the reconstruction and measurement of non-cooperative targets [1,2,3,4,5,6,7,8,9,10,11] or attitude measurement of long symmetric targets based on binocular or multiple stereo vision. There are many kinds of non-cooperative targets to be reconstructed, especially targets in the space, such as satellites and spacecrafts. Many of them are based on feature points [2], point clouds [3,4], ellipses, circles [5,6] and line structures [7,8], and so on. However, the number of the feature points is huge, and the matches between them are more complicated and less robust than the feature lines. The ellipses and circles are more difficult to extract than the feature lines. There always are obvious line features on the target. After the corresponding line matching, the reconstructed line can be used to reconstruct the target. 

At present, there is not much research on 3D line reconstruction. For binocular stereo vision, the plane intersection method [12] and Two Points (TPS) are the main two methods for line reconstruction. The principle of the plane intersection method is that the 3D line locates on the plane consisting of the optical center and the line segment in the corresponding image. The intersection line of the planes of the two cameras can be calculated. We can obtain a point on the line; therefore, we also call this method Direction-then-Point. TPS is a direct method for 3D line construction. It utilizes two pairs of corresponding image points on the 2D line (generally, they are the endpoints), then two 3D points on the line can be calculated, and the 3D line is uniquely determined. Obviously, this method does not utilize the information of the 2D line, and its accuracy depends on the points’ accuracy. DtP has a lower requirement for not needing corresponding points on the 2D line. Because the degeneracy of 3D lines is more severe than for 3D points, only 3D points on the baseline cannot be reconstructed, and the 3D line coplanar with the baseline cannot be reconstructed [13]; when the 3D line is close to coplanar with the baseline, the TPS can achieve higher accuracy than the DtP method. For a multiple camera system, the iterative method [13] based on minimizing the distance between the extracted line segment in the image and the line projected to the image is used. The reconstructed line is used by many other algorithms in computer vision. Many scholars analyze the influence of camera position, intersection angle, and relationship between the line and camera’s imaging plane on the accuracy of the reconstruction result of the 3D line [11,12,13,14,15,16,17,18,19,20]. For 3D line reconstruction in stereo vision, the optimal condition is that the angle between the optical axes of the two cameras is 90 degrees, and the 3D line is parallel to both imaging planes of the two cameras and perpendicular to, not coplanar with, the baseline [18]. However, because the baseline of the stereo cameras is always short relative to the target distance, the requirement for two cameras with near-vertical optical axes cannot be satisfied. At the same time, because the 3D lines on the target always form a certain structure, such as a triangle, a quadrangle, etc., the requirement that every 3D line be nearly parallel to the imaging plane cannot be satisfied too.

As can be seen from the above description, DtP only uses line information and does not need corresponding points’ information, but it has low position accuracy because it does not use the position information of the cameras. TPS uses two pairs of corresponding points; therefore, it can achieve better positional accuracy than DtP. However, it is difficult to obtain two pairs of corresponding image points under certain conditions. Considering the above situation, we proposed a novel method for line reconstruction based on a pair of corresponding image points and the 2D line direction. Firstly, we used the intersection method to obtain the 3D coordinates in the world coordinate system from the corresponding image points [21,22,23]. Secondly, we used this point as a point on the line to be solved to calculate the direction of the line. The point on the line is obtained before the line direction, thus call this method is named Point-then-Direction (PtD). The differences between the DtP, PtD and TPS methods are shown in Table 1.

The simulation results demonstrated that PtD can achieve more accurate results, both on direction and position than DtP, because it combines the information of the corresponding image points. At the same time, it can achieve a more accurate direction but less accurate position than TPS. If there is only one pair of corresponding image points on the line, the linear method proposed in this paper can achieve better results. 

## 2. Direction-then-Point Method

### 2.1. Stereo-Vision Scenario

**Figure 1:**
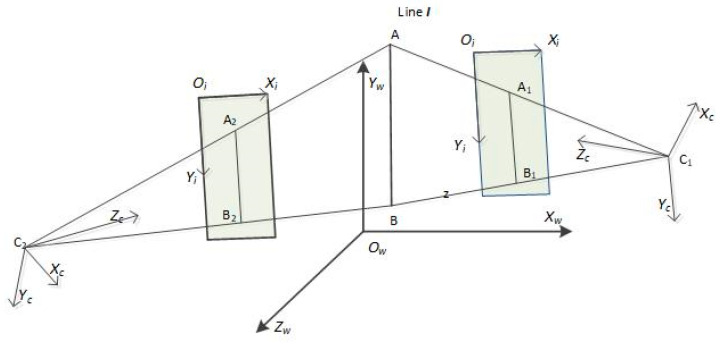
Plane intersection for stereo vision. AB is the 3D line I, C_1_ and C_2_ are the optical centers of the two cameras, A_1_B_1_ is the projection of I on camera C_1_, and A_2_B_2_ is the projection of I on camera C_2_.

A pinhole camera model [12] was used in this study. As shown in Figure 1, The world coordinate system is $O_{w}-X_{w}Y_{w}Z_{w}$, which is a right-handed coordinate system. The image coordinate system is $O_{i}-X_{i}Y_{i}$, $O_{i}$ in the top left corner of the image, $X_{i}$ faces right, and $Y_{i}$ faces down. The camera coordinate system is $O_{c}-X_{c}Y_{c}Z_{c}$, where $O_{c}$ is the camera’s optical center. The *z*-axis is pointing in the positive direction of the optical axis; the *x*-axis is perpendicular to the *z*-axis horizontally to the right; and the *y*-axis is perpendicular to the *x*-axis and to the *z*-axis, whose direction is determined by the right-handed definition criterion.

### 2.2. Plane Representation by a Single Camera

As shown in Figure 1, AB is the 3D line I, C_1_ and C_2_ are the optical centers of the two cameras, A_1_B_1_ is the projection of I on camera C_1_, and A_2_B_2_ is the projection of I on camera C_2_. When atmospheric refraction is not considered, we can assume that I is on the plane C_1_A_1_B_1_ and also on the plane C_2_A_2_B_2_. Therefore, if we can attain both of the planes’ equations in the world coordinate system, then we can obtain the direction of the 3D line and a point on the line.

Next, we derive its expression by taking a camera as an example. The equation of the line in the image coordinate system is:

(1) $$\;A^{T}X=0$$

where $A={\left[\begin{array}{ccc} a & b & c \end{array}\right]}^{T}$ and $X={\left[\begin{array}{ccc} x & y & 1 \end{array}\right]}^{T}$. The image plane is the $z_{c}=f$ plane in the camera coordinate system, where f is the physical focal length of the camera. The *z*-axis of the camera coordinate system passes through the principal point of the image plane $\left(C_{x},C_{y}\right)$, so the transformation from the image coordinate system to the camera coordinate system is determined as:

(2) $$\{\begin{array}{c} x_{c}=\left(x-C_{x}\right)d_{x}\\ y_{c}=\left(y-C_{y}\right)d_{y} \end{array}$$

where $\left(x_{c},y_{c}\right)$ is in the camera coordinate system, (x,y) is in the image coordinate system, and $\left(d_{x},d_{y}\right)$ is the actual physical size of the camera pixel.

The line in the image can be expressed in the camera coordinate system as:

(3) $$\{\begin{array}{c} a\left(\frac{x_{c}}{d_{x}}+C_{x}\right)+b\left(\frac{y_{c}}{d_{y}}+C_{y}\right)+c=0\\ z_{c}-f=0 \end{array}\;$$

The equivalent focal length of the camera is abbreviated as:

(4) $$\{\begin{array}{c} F_{x}=f/d_{x}\\ F_{y}=f/d_{y} \end{array}$$

The plane equation can be summarized as:

(5) $$F_{x}x_{c}+bF_{y}y_{c}+\left(aC_{x}+bC_{y}+c\right)z_{c}=0$$

Given the parameter matrix $n={\left[\begin{array}{ccc} aF_{x} & bF_{y} & aC_{x}+bC_{y}+c \end{array}\right]}^{T}$, the plane equation is then rewritten as:

(6) $$n^{T}P_{c}=0$$

where $P_{c}={\left[\begin{array}{ccc} x_{c} & y_{c} & z_{c} \end{array}\right]}^{T}$ is a point from the camera coordinate system, and n is the normal vector of the plane. R and t are the rotation matrix and the translation vector from the world coordinate system to the camera coordinate system, respectively. Therefore, the point in the world coordinate system can be transformed to the camera coordinate system using Equation (7).

(7) $$P_{c}=RP_{w}+t$$

where $P_{w}={\left[\begin{array}{ccc} x_{w} & y_{w} & z_{w} \end{array}\right]}^{T}$ is a point in the world coordinate system. The plane equation in the world coordinate system can then be derived as:

(8) $$n^{T}RP_{w}+n^{T}t=0$$

### 2.3. Plane Intersection

The plane equations of the two cameras, respectively, are:

(9) $$\{\begin{array}{c} n_{1}^{T}P_{w}+d_{1}=0\\ n_{2}^{T}P_{w}+d_{2}=0 \end{array}$$

The 3D line I=(l,m,n) must be located on these two planes. Therefore, the direction vector of I can be solved as:

(10) $$I=n_{1}^{T}\times n_{2}^{T}=\;\left[\begin{array}{ccc} i & j & k\\ n_{11} & n_{12} & n_{13}\\ n_{21} & n_{22} & n_{23} \end{array}\right].\;$$

A 3D line can be uniquely determined by a point and the direction vector. Therefore, we chose the foot point $P_{0}\left(x_{0},y_{0},z_{0}\right)$ from the $O_{w}$ to the I; $\left(l,m,n\right)*P_{0}{}^{T}=0$. $P_{0}$ can be solved by $\left(l,m,n\right)*P_{0}{}^{T}=0$ and Equation (9). The point is directly solved by direction, and as a result the overall accuracy mainly depends on the accuracy of the line direction.

## 3. Point-then-Direction Method

### 3.1. Stereo Vision Scenario for PtD

As shown in Figure 1, Camera C_1_ and Camera C_2_ observe the line I from different directions, and the projections on the image are A_1_B_1_ and A_2_B_2_, respectively. In this paper, we assume that one of the two endpoints of the line segments are the corresponding image points, i.e., A_1_ and A_2_ are the corresponding image points, or B_1_ and B_2_ are the corresponding image points. If the camera parameters and the line extracted from the image are error-free, the 3D point will be on the line obtained by the DtP, and then the results obtained by the DtP and Ptd are same. However, if either the camera parameters or the line extracted from the image have an error, the 3D point may not be on the line I.

**Figure 2:**
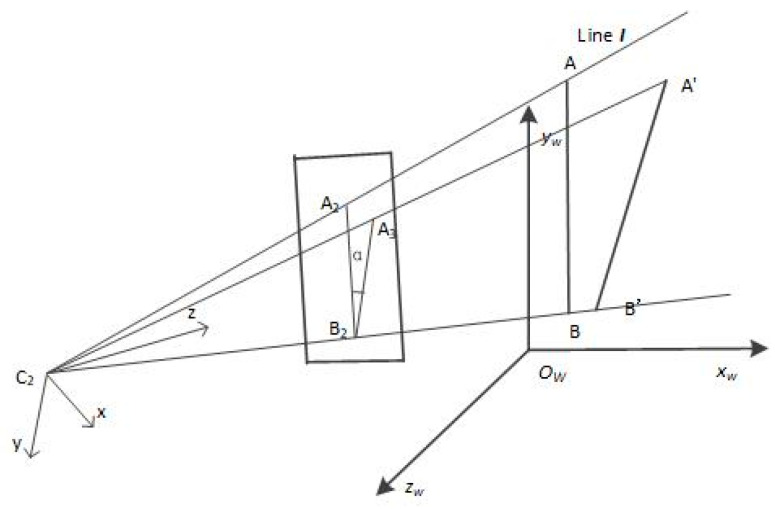
Schematic diagram of the image angle residual (IAR). The C_2_A_2_B_2_ plane is the plane composed of the optical center and the axis of the camera; AB is the 3D line; A′B′ is the reconstructed result of the 3D line; B_2_A_3_ is the projection of A′B′ on the image plane; and α is the IAR.

In a similar manner to the image-space residual method for camera calibration, the line I was projected to the image plane according to the theoretical model, and the angle between the projection and the 2D line extracted on the image plane was considered as the angle residual. We also minimized the square sum of all the angles. In this paper, this term is called the IAR. As shown in Figure 2, the C_2_A_2_B_2_ plane is the plane composed of the optical center and the axis of the camera; A’B’ is the 3D line; B_2_A_3_ is the projection of A’B’ on the image plane; and α is the image angle residual.

From the above definition and stereo-vision scenario, we can ascertain that the 3D line passes through the 3D point, and the intersection of the plane composed of the 3D line and the camera’s optical center and the corresponding imaging plane is parallel to the 2D line on the image. For a multiple camera system, we can obtain the least square solution.

The mathematical expression is derived below.

### 3.2. Image-Space Angle Residual

#### 3.2.1. Definition of the Problem

It is assumed that the direction of the 3D line  I is (l,m,n), and I passes through a point $P_{0}\left(x_{0},y_{0},z_{0}\right)$ in the world coordinate system. It is easy to see that I also passes through the point $P_{1}\left(x_{0}+l,y_{0}+m,z_{0}+n\right)$. In this paper, the $P_{0}$ is obtained by the intersection method of minimizing the distance from the spatial point to the back projection ray to obtain the three-dimensional coordinates from the corresponding image points **$A_{1}$** and **$A_{2}$** [24]. The plane of each camera passes through the point $P_{0}$,$\;P_{1}$ and the corresponding optical center. The plane consisting of  I and the optical center of the *i*th camera can be expressed as:

(11) $$\left|\begin{array}{cccc} x & y & z & 1\\ Ox_{i} & Oy_{i} & Oz_{i} & 1\\ x_{0} & y_{0} & z_{0} & 1\\ x_{0}+l & y_{0}+m & z_{0}+n & 1 \end{array}\right|=0$$

Or

(12) $$\left|\begin{array}{cccc} x & y & z & 1\\ Ox_{i} & Oy_{i} & Oz_{i} & 1\\ x_{0} & y_{0} & z_{0} & 1\\ l & m & n & 0 \end{array}\right|=0$$

where $\left(Ox_{i},Oy_{i},Oz_{i}\right)$ is the optical center of the *i*th camera. The plane equation after simplification is:

(13) $$n_{wi}{}^{T}P_{w}+d_{i}=0$$

It can be seen from Equation (12) that $n_{wi}$ can be represented linearly by I. Letting the relationship matrix be *M*:

(14) $$n_{wi}{}^{T}=I^{T}M_{i}$$

It is obvious that M is a full rank matrix. Assuming the transformation matrix from the world coordinate system to the camera coordinate system is $\left[\begin{array}{cc} R_{i} & t_{i}\\ 0 & 1 \end{array}\right]$, the plane is expressed in the camera coordinate system as:

(15) $$n_{ci}{}^{T}=n_{wi}{}^{T}\left[\begin{array}{cc} R_{i} & t_{i}\\ 0 & 1 \end{array}\right]$$

From the image plane equation z=f, the direction of the intersection line is $I_{i}=\left(n_{ci1},n_{ci2},0\right)$. Then, the angle between the 2D line in the image plane and the projection of I on the corresponding image is:

(16) $$\alpha_{i}={cos}^{-1}\left(\frac{\left[\begin{array}{cc} n_{ci1} & n_{ci2} \end{array}\right]{\left[\begin{array}{cc} a_{i} & b_{i} \end{array}\right]}^{T}}{\begin{array}{cc} \|n_{ci1} & n_{ci2}\| \end{array}\begin{array}{cc} \|a_{i} & b_{i}\| \end{array}}\right)$$

where $a_{i}$ and $b_{i}$ are coefficients of the linear equation $a_{i}x+b_{i}y+c_{i}=0$ in the image plane.

Therefore, the optimized objective function is:

(17) $$min\overset{N}{\underset{i=1}{\sum }}\alpha_{i}^{2}$$

where *N* is the number of cameras.

#### 3.2.2. Linear Method

A method of linearly solving for the minimum value is given by the derivation below.

The original expression is very complicated, especially the partial derivative expression. The numerator and the denominator contain (l,m,n), which cannot be solved linearly. Therefore, the expression is deformed.

If the two 2D lines $I_{1}={\left(a_{1},b_{1},c_{1}\right)}^{T},I_{2}={\left(a_{2},b_{2},c_{2}\right)}^{T}$ are nearly parallel, the angle is:

(18) $$\alpha_{i}={tan}^{-1}a_{1}/b_{1}-{tan}^{-1}a_{2}/b_{2}$$

This is a small angle, and we have:

(19) $$\left|\alpha_{i}\right|\approx \left|tan\left(\alpha_{i}\right)\right|=\left|\frac{\frac{a_{1}}{b_{1}}-\frac{a_{2}}{b_{2}}}{1+\frac{a_{1}a_{2}}{b_{1}b_{2}}}\right|=\left|\frac{a_{1}b_{2}-a_{2}b_{1}}{a_{1}a_{2}+b_{1}b_{2}}\right|$$

For the sake of discussion, we divided $a_{1},b_{1}$ and $a_{2},b_{2}$ by the larger one, respectively, so that the maximum value was 1. Then:

(20) $$1\leq \left|a_{1}a_{2}+b_{1}b_{2}\right|\approx \left|a_{1}{}^{2}+b_{1}{}^{2}\right|\leq 2$$

where $\left|\alpha_{i}\right|$ is positively correlated with $\left|a_{1}b_{2}-a_{2}b_{1}\right|$. |α| is the absolute value of α. Then, Equation (17) can be used instead of Equation (21) as the optimization solution objective function:

(21) $$min(\overset{N}{\underset{i=1}{\sum }}{\left(a_{i}n_{ci2}-b_{i}n_{ci1}\right)}^{2}$$

From the previous derivation, $n_{ci1},n_{ci2}$ is a linear function of $I={\left(l,m,n\right)}^{T}$. The equation can therefore be recorded as $\sum_{i=1}^{N}{\left(G_{i}{}^{T}I\right)}^{2}$, where $G_{i}$ is the coefficient of I in Equation (18), and it can be representative of a normal vector to a plane.

This can be thought of as a function of ${\left(l,m,n\right)}^{T}$, which is a function of (l,m,n). Clearly, this function is a basic elementary function—it is continuous and differentiable in the domain of the definition, and the minimum value must exist. At the point where the minimum value is obtained, the partial derivative of (l,m,n) exists and it is 0, and the partial derivative is obtained separately.

(22) $$\{\begin{array}{c} y_{l}^{\prime }=\overset{N}{\underset{i=1}{\sum }}\left(G_{i1}G_{i}{}^{T}I\right)=0\\ y_{m}^{\prime }=\overset{N}{\underset{i=1}{\sum }}\left(G_{i2}G_{i}{}^{T}I\right)=0\\ y_{n}^{\prime }=\overset{N}{\underset{i=1}{\sum }}\left(G_{i3}G_{i}{}^{T}I\right)=0 \end{array}\;.$$

We can solve Equation (19) by the SVD [25] decomposition of the coefficient matrix to obtain (l,m,n).

#### 3.2.3. Efficiency Analysis

The linear method for minimizing the image angle residual is as follows.

1. Solve the 3D coordinate of the corresponding image points with a time complexity of O(n);
2. Solve the line parameters projected onto the image plane with a time complexity of O(n);
3. Solve the coefficient matrix with a time complexity of O(n);
4. Find the final result in fixed time.

It can be seen from the above analysis that the image angle residual method can be solved only by calculating the 3D point coordinate, the projection line parameter, the solution coefficient matrix, and the least-squares solution, so the time complexity is O(n).

## 4. Experiments

Because the ground truth was not easy to obtain in the real experiment, we used the simulation to verify the accuracy of the algorithm and then we used only the real data to verify the validity of the algorithm.

### 4.1. Simulation

We simulated many conditions to validate the accuracy of our method. Firstly, the variation of the intersection angle of the two cameras and the 3D line’s attitude variation were simulated to verify the accuracy and robustness of the proposed algorithm. Secondly, the various error conditions for the stereo vision were simulated, including the 2D line extraction error, the external camera parameter error, and both the above errors. Thirdly, we verified the accuracy while in a multiple camera system. Finally, we assessed the time performance of our method in a multiple camera system.

#### 4.1.1. Simulation Environment

The simulation platform was Windows 10 Pro, the implementation language of the algorithm was C++, the implementation environment was Microsoft Visual Studio 2013, and the processor was an Intel(R) Core TM i7-9850H 2.6GHz.

**Figure 3:**
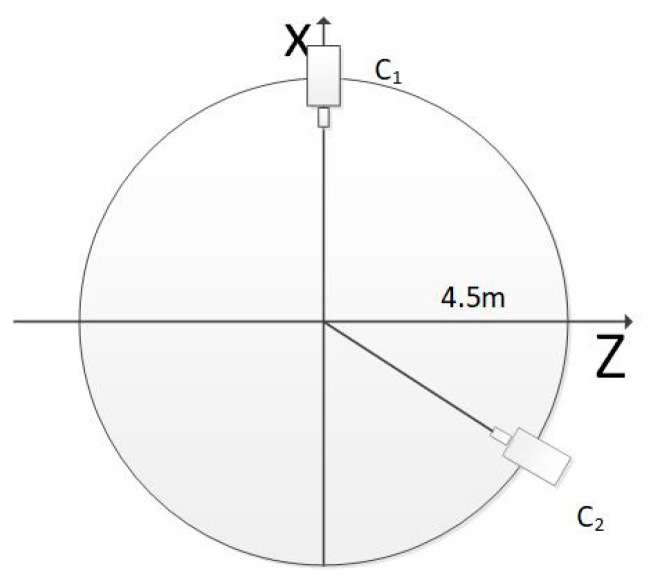
The top view of the simulation scenario.

The equivalent focal length was (2181.8, 2181.8) and the image principal point was (1024, 1024). The cameras were arranged in a circle around the target, and the radius of the circle was 4.5 m. The origin of the world coordinate system was the center of the circle. The two endpoints of the 3D line were A(−0.5,−0.5,−0.5) and B(1.5, 1.5, 1.5). The top view of the simulation scenario is shown in Figure 3.

#### 4.1.2. Simulation Description

1. The corresponding image points used in the PtD method were obtained by adding the same error to the projection of the endpoint A in the image, which ensured that PtD method was also solved under the same error conditions.
2. The TPS, DtP, and PtD methods were used for each simulation. We used the angle between the result and the ground truth and the distance from the two endpoints to the result line to evaluate the accuracy. All simulations were calculated 1000 times, and the root mean square (RMS) of the angular error and the average of distance were obtained. The units of angular error were degrees, and the units of distance error were meters.
  (23) $$E=\cos^{-1}\frac{II_{t}}{\left\|I\|\|I_{t}\right\|}$$
  (24) $$E_{d}=abs\left(d_{AI}\right)+abs\left(d_{BI}\right)$$

In the above equation $I_{t}(l_{t},m_{t},n_{t})$ is the ground truth, and I(l,m,n) is the algorithm’s result. $d_{AI}$ is the distance between the endpoint A of the 3D line segment to the result line, and $d_{BI}$ is the distance between the endpoint B of the 3D line segment to the result line.

Simulations were conducted separately to determine the following conditions:

1. Angle between optical axes of binocular cameras.
2. 3D line’s attitude.
3. Line segments extraction error in image.
4. Errors of all camera external parameters.
5. All camera external parameters and extraction errors.
6. Number of cameras.
7. Running time.

Case 1 was to verify the precision under the different angle between the two cameras. Case 2 was to verify the precision under the target’s different attitude. Cases 3–5 were to verify the precision under the error of the cameras’ parameters. Case 6 was to verify the precision as the number of cameras increased. Case 7 was to test the temporal performance of the algorithm.

#### 4.1.3. Simulation Results

The simulation details and results are given, respectively.

**Figure 4:**
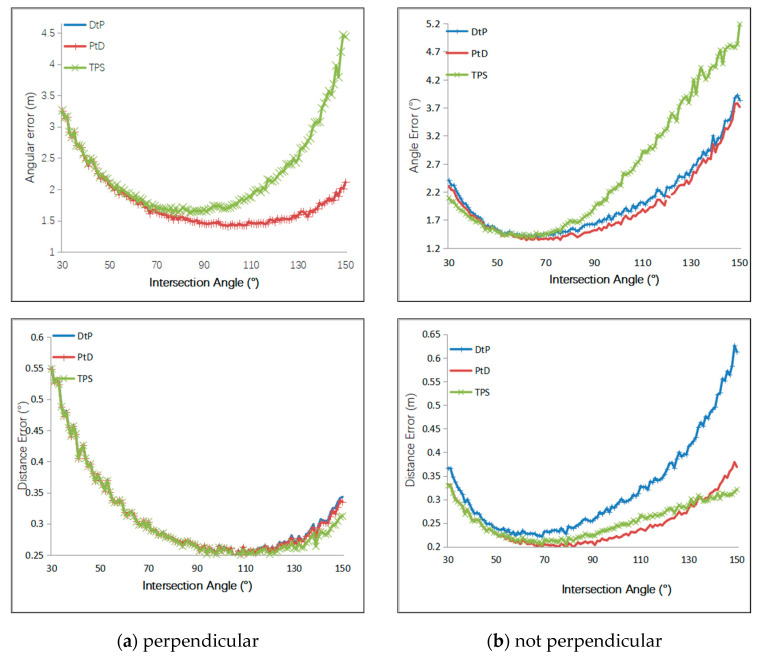
Relationship between the angular and positioning error and the intersection angle. (**a**) The result when the 3D line was parallel to the imaging plane and perpendicular to the baseline; and (**b**) the result when the 3D line was not parallel to the imaging plane and was not perpendicular to the baseline.

Firstly, we simulated the intersection angles of two cameras, which varied to verify the accuracy of the three methods. As shown in Figure 3, C_1_ was placed in the fixed point (4.5,0,0), and C_2_ was moved on the circle with a radius of 4.5 m. The angle between the optical axes of C_1_ and C_2_ varied from 30° to 150°. We simulated two conditions of the 3D line, one of which was the 3D line perpendicular to the baseline of C_1_ and C_2_. When the 3D line was perpendicular to the baseline, the endpoints of the 3D line segment were A(−0.5,−0.5,−0.5) and B(−0.5,1.5,−0.5). From the definition of the simulation scenario, we knew that, as the intersection angle changed from 30° to 150°, the line segment AB was always perpendicular to the baseline, and the distance between AB and the baseline became smaller and smaller. When the 3D line is not perpendicular to the baseline, the endpoints of the 3D line segment were A(−0.5,−0.5,−0.5), B(1.5,1.5,1.5). A Gaussian error with a mean of 0 was added to the optical center of the camera, with a 5 cm RMS value. A Gaussian error with a mean of 0 was added to the camera angle, with a 1° RMS value. A Gaussian error with a mean of 0 was added to the *x*- and *y*-directions of the two endpoints of the 2D line segments, with a 2-pixel RMS value. The results are shown in Figure 4.

When the 3D line remained unchanged, the error was relevant to the intersection angle. In the beginning, the three methods achieved the same accuracy. As the intersection angle varied from 30° to 150°, the distance between the baseline and AB grew smaller and smaller. In other words, they were becoming closer and closer to each other, which meant the intersection condition was getting worse; the PtD method had the same accuracy as the DtP method. TPS had the best positional accuracy but the worst angular accuracy. In the condition where the 3D line was not parallel to the imaging plane and was not perpendicular to the baseline, PtD had higher accuracy both on the position and angle than DtP; TPS achieved the worst angular accuracy, and DtP achieved the worst positional accuracy.

**Figure 5:**
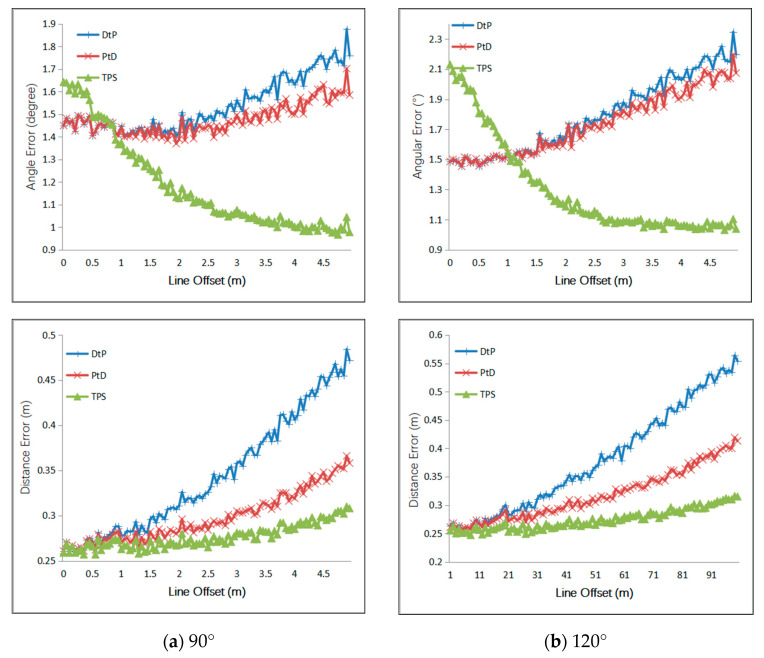
Relationship between the angular and positioning error and the line offset. (**a**) The result when the intersection angle was 90°; and (**b**) the result when the intersection angle was 120°.

Secondly, we simulated the condition where the 3D line moved while the intersection angle remained unchanged; the C_1_ and C_2_ places were fixed. Endpoint A was (−0.5,−0.5,−0.5), The initial position of endpoint B was (−0.5,1.5,−0.5), and the kth position was (−0.5−k∗0.05,1.5,−0.5−k∗0.05); k was the offset time. The end position of point B was (−5.5,1.5,−5.5). A Gaussian error with a mean of 0 was added to the optical center of the camera, with a 5 cm RMS value. A Gaussian error with a mean of 0 was added to the camera angle, with a 1° RMS value. A Gaussian error with a mean of 0 was added to the *x*- and *y*-directions of the two endpoints of the 2D line, with a 2-pixel RMS value. The results of the simulation while the intersection angles are 90° and 120°, and shown in Figure 5a,b respectively.

As shown in Figure 5, at the start, the TPS method had the best positioning accuracy and the worst angular accuracy. However, as the intersection condition became, the TPS had the best accuracy both in direction and position. The reasons are as follows.

Firstly, the 3D line became longer, and the final length was 3.68-times greater than that of the initial length. Therefore, theoretically, if only the influence of the line length was considered, the direction error of TPS would be the 1/3.68 of the original value. However, the error of the 3D point B increased because the intersection angle of 3D point B became smaller. Therefore, the error reduction was not proportional.

Secondly, the angle between the 3D line and the baseline remained unchanged—it was always 90°. However, the angle between the 3D line and the imaging plane varied from 0 to 45° and the distance between the 3D line and the baseline became smaller. At the start, when the intersection angle was 90°, the distance between the 3D line and the baseline was 3.89 m; when the intersection angle was 120°, the distance between the 3D line and the baseline was 2.94 m. At the end, however, the distances were 2.50 m and 2.30 m, respectively. The lengths of the baseline were 6.36 m and 7.79 m, respectively. From the above analysis, we can determine that the smaller distance aggravated the degeneracy of the lines’ reconstruction.

**Figure 6:**
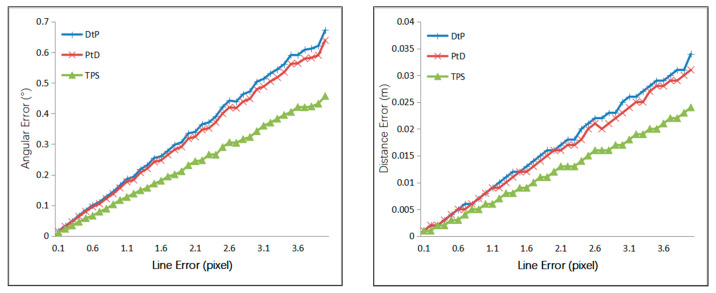
Relationship between the line error and error of 2D line.

Thirdly, we simulated the condition where the cameras and the 3D line remained unchanged, but the errors of the camera’s external parameters and 2D line were different. The intersection angle was 120°. The endpoints of the 3D line were A(0.5,−0.5,0.5) and B(1.5,1.5,1.5). We simulated for 2D line error, the error of the camera external parameters, and all above errors. The error details were as below: 

Case 1: A Gaussian error with a mean of 0 was added to the *x*- and *y*-directions at the head and the tail of the line segment, with 0–4 pixels of RMS. The results are shown in Figure 6.

From Figure 6, we can see that if only the same 2D line error existed, the 3D line’s accuracy of the three methods, from most to least accurate, was TPS, PtD, then DtP.

**Figure 7:**
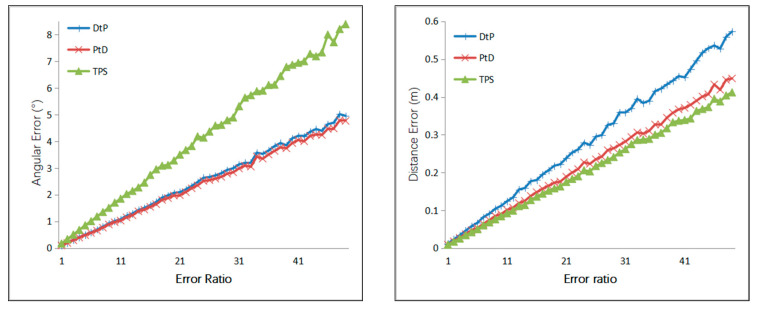
Relationship between the line error and error of external camera parameters.

Case 2: A Gaussian error with a mean of 0 was added to the optical center of the camera, with 1 mm to 50 mm of RMS, and the step size was 1 mm. A Gaussian error with a mean of 0 was added to the camera angle, with 0.05–2.5° of RMS. The simulation results are shown in Figure 7.

From Figure 7, we can see that under the same error of the camera’s external parameters, the directional accuracy of the 3D line of the three methods, from most to least accurate, was PtD, DtP, then TPS, and the positional accuracy of the 3D line of the three methods from most to least accurate, was TPS, PtD, then DtP.

**Figure 8:**
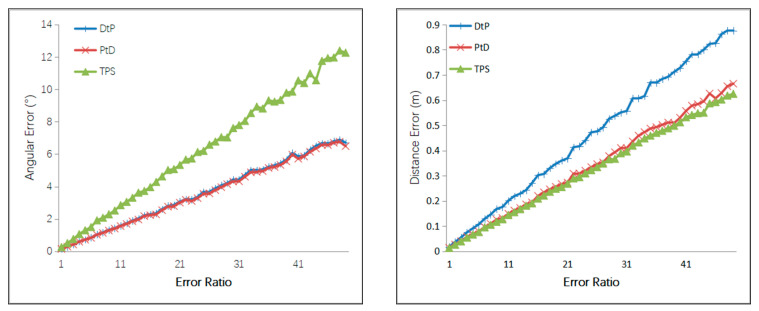
Relationship between the line error and the error of intersection parameters.

Case 3: A Gaussian error with a mean of 0 was added to the optical center of the camera, with 1 mm to 50 mm of RMS, and the step size was 1 mm. A Gaussian error with a mean of 0 was added to the camera angle, with 0.05–2.5° of RMS. A Gaussian error with a mean of 0 was added to the *x*-and *y*-directions of the two endpoints of the 3D line, with 0.1–5 pixels of RMS. The simulation results are shown in Figure 8.

From Figure 8, we can see that under same error of external camera’s parameters and the 2D lines, the 3D line’s direction accuracy of the three methods, from most to least accurate, was PtD, DtP, then TPS, and the 3D line’s position accuracy of the three methods, from most to least accurate, was TPS, PtD, then DtP. The position accuracies of TPS and PtD are close, and the directional accuracy of PtD was far better than TPS. 

**Figure 9:**
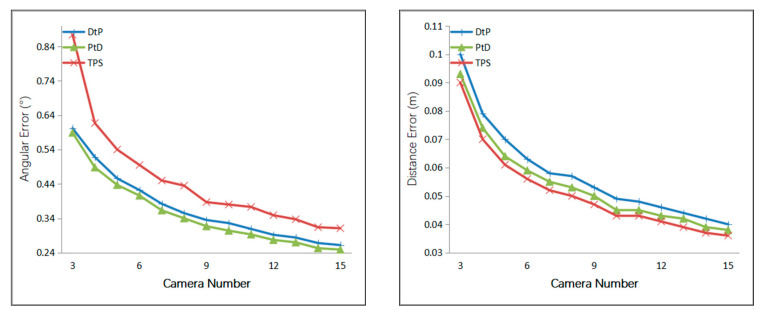
Relationship between the line error and the camera number.

Additionally, we simulated the condition where the camera number varied from 2 to 20; the cameras were evenly distributed on the circle as shown in Figure 3. The DtP method used the LS [26] directly solve this scenario. A Gaussian error with a mean of 0 was added to the optical center of the camera, with a 10 mm root mean square (RMS) value. A Gaussian error with a mean of 0 was added to the camera angle, with a 0.5° RMS value. A Gaussian error with a mean of 0 was added to the *x*- *y*-directions of the two endpoints of the 3D line, with a 1-pixel RMS value. The endpoints of the 3D line were A(0.5,−0.5,0.5),B(1.5,1.5,1.5). The results are shown in Figure 9.

From Figure 9, we can see that as the number of cameras increased, the accuracy of the three methods increased gradually, indicating that these methods utilized the constraints of the multi-camera. Similar to the result in Figure 8, with the increase in the number of cameras, under the same error of the external camera’s parameters and the 2D lines, the 3D line’s direction accuracy of the three methods, from most to least accurate, was PtD, DtP, then TPS, and the 3D line’s positional accuracy of the three methods, from most to least accurate, was TPS, PtD, then DtP. The positional accuracies of TPS and PtD were close, and the directional accuracy of the PtD method was far better than the TPS method.

**Figure 10:**
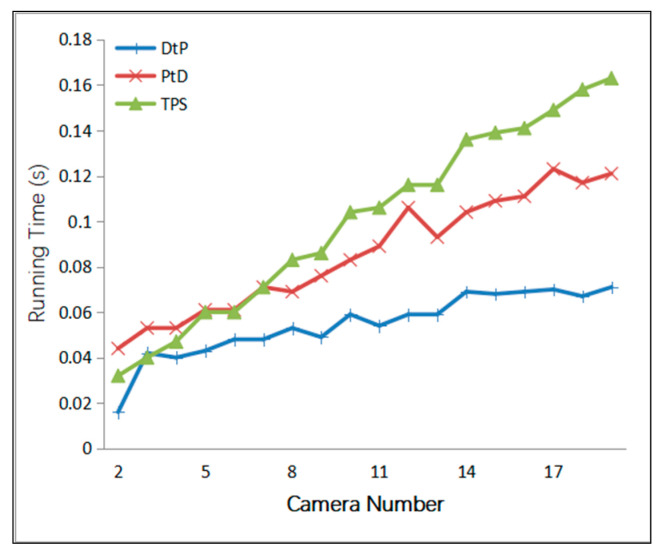
Relationship between the running time (in seconds) and the number of cameras. TPS, PtD, and DtP are linear methods, but PtD takes longer than DtP.

Finally, we simulated the condition where the camera number increased from 2 to 20 to verify the time performance of our method. The linear algorithm took a small amount of time; therefore, for the convenience of comparison, we counted the time it took to run 10,000 times. A Gaussian error with a mean of 0 was added to the optical center of the camera, with a 5 cm RMS value. A Gaussian error with a mean of 0 was added to the camera angle, with a 1° RMS value. A Gaussian error with a mean of 0 was added to the *x*- *y*-directions of the two endpoints of the 2D line segments, with a 2-pixel RMS value. The simulation results are shown in Figure 10.

Figure 10 confirms that all three methods are linear methods; TPS took the longest, then PtD, and the fastest was DtP. DtP only needs to solve each plane equation and then solve the least-squares method; PtD needs to solve the 3D point coordinate and the line parameters in two steps; TPS needs to solve the 3D point coordinate two times, which explains the variances in running time between the methods.

#### 4.1.4. Simulation Conclusion

The simulation results are shown in Table 2. The “F” in the table means first, the “S” means second, and the “T” means third and the “S->T” means from second to third.

From the above simulation results, we can come to some conclusions:

1. Under the same error of the external camera parameters and the 2D line, the PtD method has the best directional accuracy, and the TPS method has the best positional accuracy.
2. When the intersection condition was normal, the 3D line’s direction accuracy of the three methods, from most to least accurate, was PtD, DtP, then TPS, and the 3D line’s position accuracy of the three methods, from most to least accurate, was TPS, PtD, and DtP. Therefore, if the direction accuracy is more important, the PtD method should be used, and if positional accuracy is more important, the TPS method should be used—provided that there are two pairs of corresponding image points.
3. When the intersection condition was bad and the target was relative long, TPS achieved the best accuracy, both in the direction and position of the 3D line. However, it needs two pairs of corresponding image points, which is sometimes hard to satisfy. PtD achieved better accuracy than DtP both in the direction and position of the 3D line, so PtD can be used if there is only one pair of corresponding image points.

### 4.2. Physical Experiment

We used physical experiment 1 to verify the precision of our method, and used physical experiment 2 (in which TPS could not be used) to verify the correctness of the algorithm.

#### 4.2.1. Physical Experiment 1

##### Physical Environment

**Figure 11:**
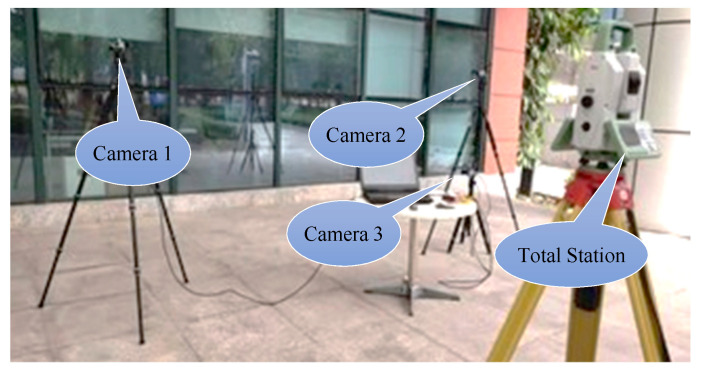
Physical scene for experiment 1.

**Figure 12:**
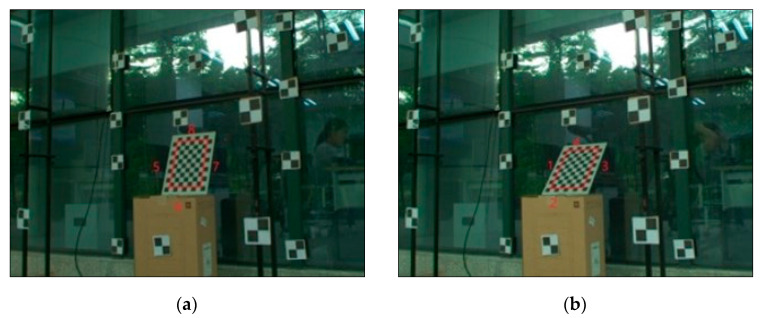
The image captured by camera 1. The eight 3D lines to be reconstructed are marked by the red lines on the images; (**a**) is more perpendicular and in a better intersection condition than (**b**).

We used three cameras to measure the lines—the camera setup is shown in Figure 11. The physical environment is shown in Figure 12. The target was a checkerboard and was placed in front of the cameras. The precision of the checkerboard was 0.01 mm. The 3D lines to be reconstructed were the four edge lines in the checkerboard, as marked in Figure 12. The length of the lines 1, 3, 5, and 7 were 30 cm, and the lines 2, 4, 6, 8 were 21 cm. From the positional relationship between the camera and the target, we could ascertain that (a) was in a better intersection condition than (b), and the lines 1 and 3 were in a better intersection condition than the lines 2 and 4. All cameras were calibrated by the diagonal markers behind the checkerboard, and the 3D coordinates of the diagonal markers were obtained by the total station Leica TS60, with an accuracy of 0.6 mm. At the same time, we also obtained the 3D coordinates of the four corners of the checkerboard using the Leica TS60. PtD, DtP, and TPS methods were used to reconstruct the four 3D lines. The camera and lens models were Basler (acA1440—220uc) and RICOH (6 mm 1:1.4), respectively.

##### Experimental Procedure

The procedure of the experiment was as follows:

1. The world coordinate system was established by the total station. The total station was used to obtain the coordinates of the calibration control points. The coordinate system direction was vertically upwards (*Y*) and horizontal (*XZ*), and the system constituted a right-hand coordinate system.
2. The stereo cameras were used to take photos synchronously. The checkerboard was placed at two angles to verify the accuracy of the methods in two conditions. One was where the 3D lines were parallel to the imaging plane, and the other was not. The calibration control points were extracted from the image, and the 3D coordinates were used to calibrate all of the cameras in a unified world coordinate system. All parameters of the camera were calibrated, including the main point, equivalent focal length, lens distortion, and external parameters of the camera.
3. The 2D lines and the corresponding image points were extracted from the two images by extracted the two endpoints of the 2D lines. The corresponding image points were one of the endpoints of the 2D lines.
4. After the correction of lens distortion, DtP, PtD, and TPS were used separately to obtain the 3D line ***I***.

First, we estimated the precision by the distance of two points—the results are shown in Table 3. From the results, we determined that the maximum of the distance error was 0.2 mm and the corresponding angle was ${tan}^{-1}\left(\frac{0.2}{300}\right)=0.038{}^{\circ}$. The theoretical accuracy of the total station was 0.6 mm, which meant a corresponding angle ${tan}^{-1}\left(\frac{0.6}{210}\right)=0.16{}^{\circ}$.

The errors between the coordinates obtained by the total station and given by the intersection method [21] to demonstrate the precision of the cameras’ parameters are shown in Table 4. The *i*th point was the top or left endpoint of the *i*th line.

Therefore, we used the point coordinates obtained by the total station to calculate the direction of the line as the ground truth $I_{t}$. We calculated the angle between $I_{t}$ and I as the angular error, and the sum of distances from the two endpoints to I as the distance error, to estimate the accuracy of our method.

All the results are shown in Table 5 and Table 6. The second, third and fourth columns are angular errors (°), and the fifth, sixth and seventh columns are distance errors (mm). 

From the above tables, we can see that for the lines 1, 3, 5, and 7, the sum of the angular errors of the 3D line, from lowest to highest, was TPS, PtD, then DtP, and they were very close; the maximum difference was 0.02. The sum of the distance error of the 3D line, from highest to lowest, was DtP, PtD, then TPS. For the lines 2, 4, 6, and 8, the accuracy of the direction and distance, from lowest to highest, was TPS, PtD, then DtP. However, the TPS needed two pairs of corresponding image points, which was difficult to meet under certain conditions, such as in physical experiment 2.

#### 4.2.2. Physical Experiment 2

Physical experiment 2 was designed to verify the correctness of our method if there were more than two cameras.

##### Physical Environment

**Figure 13:**
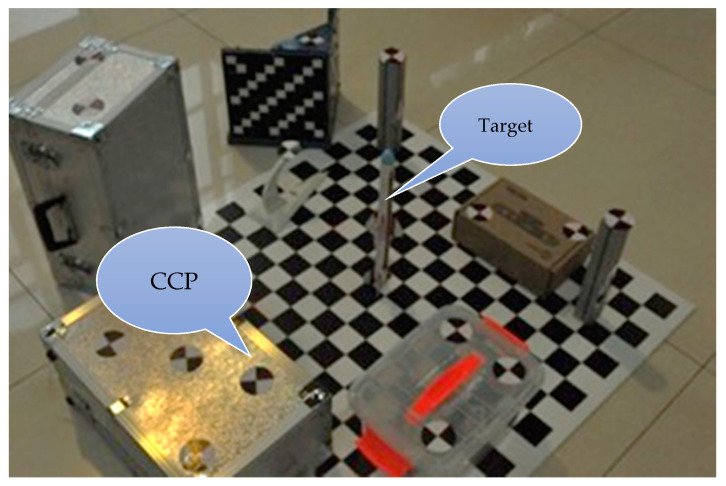
Physical scene for experiment 2. The target was placed on the board in the center, and the diagonal markers on the side were the calibration control points (CCPs). The 3D line to be reconstructed was the target’s central axis.

The setup for the physical experiment is shown in Figure 13. The target was placed on the board in the center, and the diagonal markers on the side were the calibration control points. The 3D line to be reconstructed was the target’s central axis. The resolution of the camera was 4288 × 2848 pixels, and the equivalent focal distance was approximately 5200.

##### Experimental Procedure

The experiment was conducted as follows:

1. The total station was used to obtain the coordinates of the calibration control points in the world coordinate system. The total station coordinate system was used as the world coordinate system. The coordinate system direction was vertically upwards (*Y*) and horizontal (*XZ*) and the system constituted a right-hand coordinate system.
2. The same camera was used to take photographs from nine different locations. The calibration control points were extracted from the image, and the three-dimensional coordinates were used to calibrate all of the cameras in a unified world coordinate system.
3. The 2D line was extracted from the image. The corresponding image point was the top point of the target.
4. DtP and PtD were used separately to obtain the results. For hard-to-find pairs of corresponding image points on the target, TPS was not used.

All the target directional results are shown in Table 7. It can be seen from the table that the results of the two methods were essentially the same.

## 5. Conclusions

Considering the problem of 3D line reconstruction in binocular or multiple view stereo vision, where there is only a pair of corresponding points on the line, a new method called PtD was proposed. Compared to DtP and TPS methods, PtD uses both point and line information for 3D line reconstruction. The simulation results in this paper demonstrate that our method achieves the best accuracy for determining direction; and although the positional accuracy of our method is worse than TPS, it is better than DtP. The physical experiment shows that under bad intersection conditions, the PtD method achieves better accuracy than the DtP method.

## Figures and Tables

**Table 1:** The differences between Direction-then-Point (DtP), Point-then-Direction (PtD) and Two Points (TPS) methods.

Difference	DTP	PtD	TPS
Pairs of corresponding image points	0	1	2
Can be reconstructed while the line is coplanar with the baseline (no point on the baseline)	×	×	✓
Can be reconstructed while the line is coplanar with the baseline (one point on the baseline)	×	×	×
Use of the camera’s optical center	×	✓	✓
Use of the information of the 2D line	✓	✓	×

**Table 2:** All simulation results.

Method	Case 1		Case 2		Case 3	Case 4	Case 5	Case 6
	Perpendicular	Not Perpendicular	90°	120°				
(A)DtP	S	**F**	S→T	S→T	T	S	S	S
PtD	**F**	**F**	F→S	F→S	S	**F**	**F**	**F**
TPS	T	S	**T**→**F**	**T**→**F**	F	T	T	T
DtP(D)	T	S	T	T	T	T	T	T
PtD	S	S	S	S	S	S	S	S
TPS	**F**	**F**	**F**	**F**	**F**	**F**	**F**	**F**

**Table 3:** Errors of the total station (mm).

Line No.	Measured Distance	Ideal Distance
1	300.0	300.0
2	210.1	210.0
3	300.2	300.0
4	210.0	210.0
5	300.1	300.0
6	210.1	210.0
7	300.0	300.0
8	210.0	210.0

**Table 4:** Results of 3D points’ errors (mm).

Point No.	*X*	*Y*	*Z*
1	−0.2	−0.7	−0.0
2	−0.5	−0.5	−0.1
3	−0.2	−0.6	−0.0
4	0.4	−0.5	0.8
5	−0.4	−0.2	0.6
6	−0.0	−0.0	0.1
7	−0.5	−0.6	0.2
8	−0.1	−0.5	0.7

**Table 5:** Experiment results for good conditions.

No.	DtP	PtD	TPS	DtP	PtD	TPS
1	0.10	0.10	**0.09**	2.4	**1.2**	1.4
3	**0.10**	0.11	0.12	1.6	1.5	**1.4**
5	**0.12**	**0.12**	**0.12**	0.9	0.8	**0.7**
7	**0.06**	0.07	0.08	1.6	**1.5**	1.6
sum	0.38	0.4	0.41	6.5	**5**	5.1

**Table 6:** Experiment results for bad conditions.

No.	DtP	PtD	TPS	DtP	PtD	TPS
2	0.58	0.28	**0.03**	7.7	2.0	**1.1**
4	0.78	0.50	**0.23**	6.8	2.0	**1.6**
6	**0.07**	**0.07**	0.16	2.9	**0.4**	0.7
8	1.07	1.08	**0.08**	4.1	4.3	**1.5**
sum	2.5	1.93	**0.5**	21.5	8.7	**4.9**

**Table 7:** Experimental results.

Camera No.	DtP	PtD
2	(−0.012, 1, −0.014)	(−0.012, 1, −0.014)
6	(−0.020, 1, −0.022)	(−0.016, 1, −0.019)
9	(−0.017, 1, −0.012)	(−0.018, 1, −0.014)

## Data Availability

All experimental data in this paper were created by this study.
